# Polarization optical coherence tomography optoretinography: verifying light-induced photoreceptor outer segment shrinkage and subretinal space expansion

**DOI:** 10.1117/1.NPh.12.1.015005

**Published:** 2025-01-27

**Authors:** Shaiban Ahmed, Taeyoon Son, Guangying Ma, Xincheng Yao

**Affiliations:** a University of Illinois Chicago, Department of Biomedical Engineering, Chicago, Illinois, United States; b University of Illinois Chicago, Department of Ophthalmology and Visual Sciences, Chicago, Illinois, United States

**Keywords:** intrinsic optical signal, optoretinography, optical coherence tomography, retina, photoreceptor, polarization

## Abstract

**Significance:**

Stimulus-evoked intrinsic optical signal (IOS) changes in retinal photoreceptors are critical for functional optoretinography (ORG). Optical coherence tomography (OCT), with its depth-resolved imaging capability, has been actively explored for IOS imaging of retinal photoreceptors. However, recent OCT studies have reported conflicting results regarding light-induced changes in the photoreceptor outer segments (OSs), with both elongation and shrinkage being observed. These discrepancies may stem from the difficulty in reliably identifying OS boundaries, particularly the inner segment/outer segment (IS/OS) junction and OS tip, as well as potential confusion with subretinal space dynamics. Gaining a better understanding of these light-induced OS changes is essential for accurate interpretation of ORG measurements and for optimizing IOS imaging systems to enhance sensitivity.

**Aim:**

We aim to develop a method for the reliable identification of OS boundaries and to verify light-induced photoreceptor OS shrinkage and subretinal space expansion.

**Approach:**

We employed a polarization-resolved full-field swept-source optical coherence tomography system capable of sequentially capturing parallel-polarization and cross-polarization OCT signals. The parallel-polarization mode is optimized to detect ballistically reflected photons from well-defined retinal boundaries, such as the IS/OS junction and the photoreceptor tips, whereas cross-polarization primarily captures multiply scattered photons. This differentiation enables parallel-polarization OCT to minimize the interference from scattered photons, enhancing the precision of OCT band quantification.

**Results:**

Parallel-polarization OCT revealed photoreceptor OS shrinkage and subretinal space expansion in light conditions compared with dark conditions. Moreover, the overall outer retinal length appeared to swell under light. These observations were consistently confirmed in four healthy adult human subjects.

**Conclusions:**

Parallel-polarization OCT provides a reliable method for identifying the IS/OS junction and OS tip, confirming light-induced photoreceptor OS shrinkage and subretinal space expansion.

## 1 Introduction

The retina is a neurovascular complex located in the posterior segment of the eye. It plays a critical role in capturing photons, converting light energy into bioelectric signals, and processing initial visual information. Any damage to the retina can severely impair visual function, making retinal examination essential for diagnosing eye diseases, monitoring disease progression, and assessing treatment efficacy. In addition, as part of the central nervous system (CNS), the retina provides a non-invasive window into CNS health and may serve as an indicator of neurodegenerative disorders, such as Alzheimer’s disease (AD) and other forms of dementia.^1^^,^^2^ Therefore, monitoring retinal function also holds potential for the early diagnosis and evaluation of neurodegenerative diseases.^3^^,^^4^

Electrophysiological methods such as focal electroretinography (ERG)^5^ and multifocal ERG^6^ provide an objective assessment of retinal physiological function. However, these electrophysiological signals are aggregated from the whole depth of the retina, offering limited spatial resolution. Stimulus-evoked intrinsic optical signal (IOS) changes in the retina hold promise for high-resolution functional optoretinography (ORG).^7^^,^^8^ Different optical imaging systems, such as near-infrared light microscopy,^7^ confocal imagers,^9^ fundus cameras,^10^ adaptive optics,^11^ and optical coherence tomography (OCT),^12^^–^^14^ have been previously explored for functional IOS imaging. Among these techniques, OCT can provide depth-resolved imaging of retinal IOS signals at high spatial and temporal resolution.

Functional OCT has been actively explored for dynamic imaging of stimulus-evoked IOS changes in both human and animal subjects.^12^^–^^36^ Both intensity^12^^–^^15^^,^^17^^–^^20^^,^^29^^–^^31^^,^^35^ and phase-sensitive^16^^,^^21^^,^^22^^,^^25^^,^^32^^,^^33^^,^^36^ OCT measurements have been validated for functional IOS imaging. However, different OCT studies have reported conflicting results regarding light-induced alterations of the photoreceptor outer segment (OS) length. Light-evoked elongation of photoreceptor OS has been observed when dark-adapted mouse models were exposed to prolonged light adaptation (ranging from 15 min to ∼5  h)^17^ or even brief light exposure (OS elongation was detected as early as 2 min after light exposure).^19^ In human subjects, light-induced oscillatory changes, initiated with OS elongation and followed by shrinkage within 10 to 30 min, were reported.^18^ Although these observations were derived from intensity-based OCT imaging, phase-resolved OCT has also been employed to study photoreceptor OS dynamics.^16^^,^^23^^,^^25^^,^^32^^,^^33^^,^^36^ Phase-resolved OCT revealed a small magnitude shrinkage of the OS optical path length within 10 to 15 ms of stimulation onset, followed by a larger magnitude swelling over a few seconds. By contrast, light-induced shrinkage of the photoreceptor OS equivalent length (OSEL) has been reported when continuous light exposure was given to dark-adapted healthy human subjects. This OSEL shrinkage peaked around 10 to 20 min of light-adaptation.^15^ Microscopic examination of freshly isolated rod OS and intact frog retina also revealed a light-driven shrinkage of the OS.^37^^,^^38^ Contrastingly, using the X-ray diffraction technique, light-induced elongation of the OS was observed in the isolated retina.^39^ Variations in stimulation protocols, observational time points, and challenges in precisely determining OS boundaries, particularly at the inner segment/outer segment (IS/OS) junction and OS tip, may account for these discrepancies. In addition, complexities involving subretinal space dynamics could further contribute to the observed inconsistencies.

We recently developed a polarization-resolved full-field swept-source OCT (FF-SS-OCT) system capable of both parallel-polarization and cross-polarization OCT measurements and validated it for polarization-resolved characterization of the outer retina.^40^^,^^41^ Parallel-polarization OCT excels in detecting layer-like structures, such as the inner plexiform layer (IPL) sub-layers, outer plexiform layer (OPL) sub-layers, and photoreceptor OS tips, making it particularly well-suited for precise measurement of OCT band distances. By contrast, cross-polarization OCT predominantly captures multiply scattered photons from structures such as the ellipsoid zone (EZ), OS interdigitation region, and retinal pigment epithelium/Bruch’s membrane (RPE/BM) complex, making it more adept at mapping scattering dynamics in the retina.

In this study, we employed polarization-resolved FF-SS-OCT to characterize outer retinal changes under light and dark conditions. By minimizing the impact of multiply scattered photons on OCT band quantification, parallel-polarization OCT confirmed light-driven OS shrinkage and subretinal space expansion in the human retina.

## 2 Method

### 2.1 System Design

**Fig. 1:**
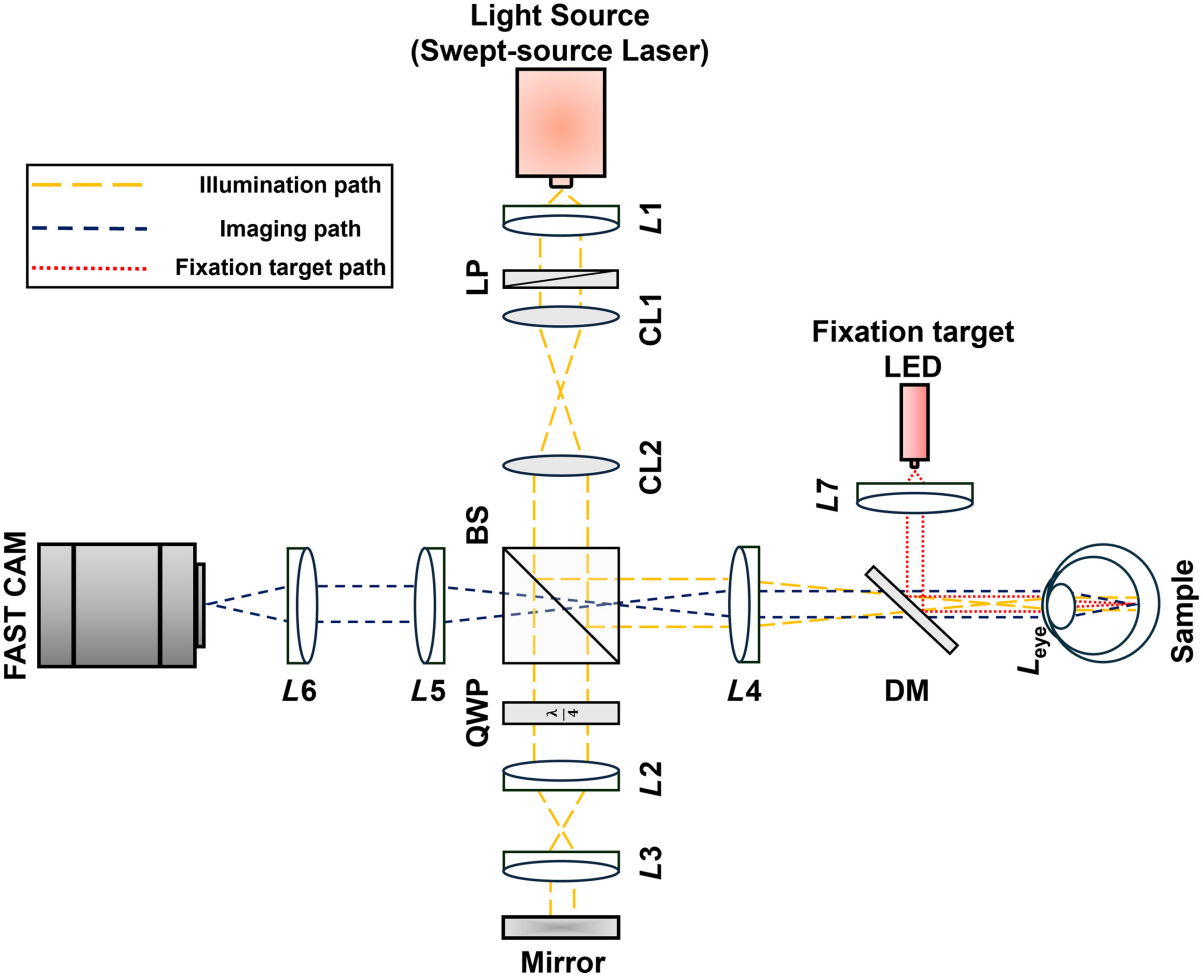
Schematic representation of the FF-SS-OCT system. L1−L7, achromatic lens; BS, beam splitter; CL, cylindrical lens; DM, dichroic mirror; LP, linear polarizer; QWP, quarter waveplate.

A custom-designed polarization-resolved FF-SS-OCT system (Fig. 1) was used for this study. Technical details of this system have been reported in our recent publication.^40^ Briefly, the system employs a tunable swept-source laser (λ=840  nm, Δλ=75  nm; Broad Sweeper BS-840-HP-1, SUPERLUM Inc., Carrigtwohill, Co. Cork, Ireland) to illuminate the retina with an illumination power of 2.5 mW. An achromatic collimator (L1) was used to collimate the illumination light, which was then passed through a linear polarizer (LP). Two cylindrical lenses (CL1 and CL2) were used to expand the light beam in one dimension. A beam splitter split the linearly polarized light into sample and reference beams. The reference beam was directed to a mirror, whereas the sample beam was focused onto the external focal plane of the eye lens to achieve parallel illumination at the retinal plane. The backscattered light from the retina interfered with the reflected light from the reference mirror to form the OCT signal. A quarter waveplate (QWP) was positioned in the reference arm to control the polarization state of the reference beam. For the parallel-polarization mode, the QWP’s fast axis was aligned parallel (0 deg) to the polarization axis of the incident light. In the cross-polarization mode, the QWP was rotated so that its fast axis was aligned at 45 deg to the polarization axis, creating a perpendicular (90 deg) alignment in the reflected beam path. Imaging was performed using a high-speed camera (FASTCAM NOVA S-16, Photron, Japan), which recorded the interference signal over a central region of 1024×128  pixels (width: 1024  μm; height: 128  μm) at a frame rate of 100 kHz. The wavelength sweeping resolution is 75  nm/s, corresponding to 1000 sampling wavelengths per sweep, resulting in volumetric data dimensions of 1024×128×1000  voxels. The system achieved an axial resolution of 4.1  μm and a lateral resolution of 5.6  μm in air. The calibrated axial pixel sampling resolution was 3.8  μm in air and 2.9  μm in retinal tissue, with a lateral pixel sampling ratio of 1  μm. A 0.05-μW red LED fixation target was used to stabilize the eye during imaging, minimizing voluntary eye movements. This target was quantitatively controlled to ensure consistent imaging locations across successive recordings for reliable data collection from different nasal eccentricities. Data acquisition and system control were streamlined through a custom LabView code.

### 2.2 Data Acquisition

This study was approved by the University of Illinois Chicago Institutional Review Board and adhered to the Declaration of Helsinki’s ethical standards. Four healthy subjects without ocular diseases (age: 31±5; race: Asian) participated after providing fully informed written consent, ensuring their understanding of the experimental procedures. All experiments were conducted between 9:00 AM and 5:00 PM to avoid peak hours of disk shedding, which is most likely to be active in the early morning.^42^ Subjects were kept in ambient light before the initial imaging session, and non-mydriatic data were first recorded under light-adapted conditions. For comparative assessment, OCT data were assessed from three specific locations: 0-, 2-, and 10-deg nasal eccentricities. These locations were strategically selected to analyze the outer retinal dynamics during the light-to-dark transition across areas with varying rod and cone photoreceptor densities. For each imaging session, parallel-polarization and cross-polarization OCT data were acquired consecutively, with a 30-s QWP adjustment period between the recordings. After parallel-polarization recording, the QWP was manually rotated 45 deg using a cage rotation mount. During this adjustment, the subjects rested their chin and forehead against the chin and forehead rest while fixating their vision on the fixation target. The system alignment was kept unchanged during the 30-s QWP adjustment period, facilitating data recording from the exact location. After the light-adapted data acquisition, subjects were kept in a dark room, with the imaged eye covered by an eye patch to provide further protection from stray light. The remaining stray light was blocked by covering all the electronic devices in the imaging area with dark materials. Following 30 min of dark adaptation, subjects were re-imaged, and parallel-polarization and cross-polarization OCT data were collected again at 0-, 2-, and 10-deg nasal eccentricities from the fovea. It must be noted that two separate light and dark-adapted imaging sessions were required for each subject. For the first session, the fixation target was centered at 1 deg. The designed system has a field of view of 1024  μm×128  μm; hence, at 1 deg, we obtained data for 0- and 2-deg nasal eccentricities. To prevent potential discomfort, prolonged imaging sessions were avoided. The subject was re-imaged the following day to acquire 10-deg nasal eccentricity data, with the fixation target laterally adjusted to 10-deg using translational stages.

### 2.3 Data Processing and Statistical Analysis

**Fig. 2:**
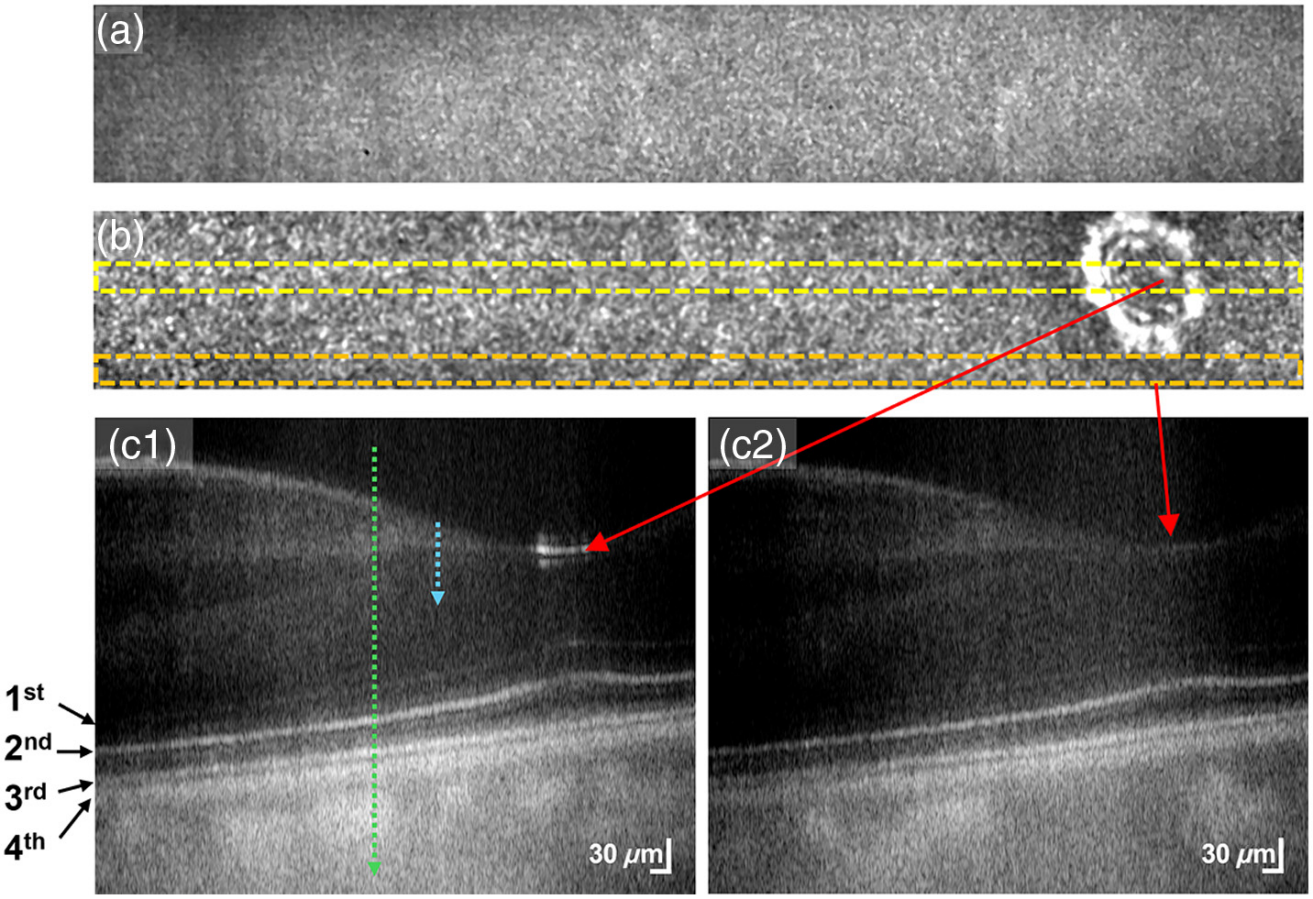
Volumetric registration using biological references illustrated in parallel-polarization data. (a) Enface image generated by mean projection across the full depth, indicated by the green arrow in panel (c1). (b) Enface image generated by mean projection from the inner retina, spanning the IPL to Henle’s fiber layer (HFL), indicated by the blue arrow in panel (c1). Notably, the central foveal reflection pattern is only visible in panel (b), making it a reliable reference marker for image registration. (c1) Averaged B-scan generated by spatially averaging 30 B-scans from the region demarcated by the yellow window in panel (b). (c2) Averaged B-scan generated by spatially averaging 30 B-scans from the region demarcated by the orange window in panel (b). These B-scans were obtained from a single volume before volumetric averaging and flattening.

**Fig. 3:**
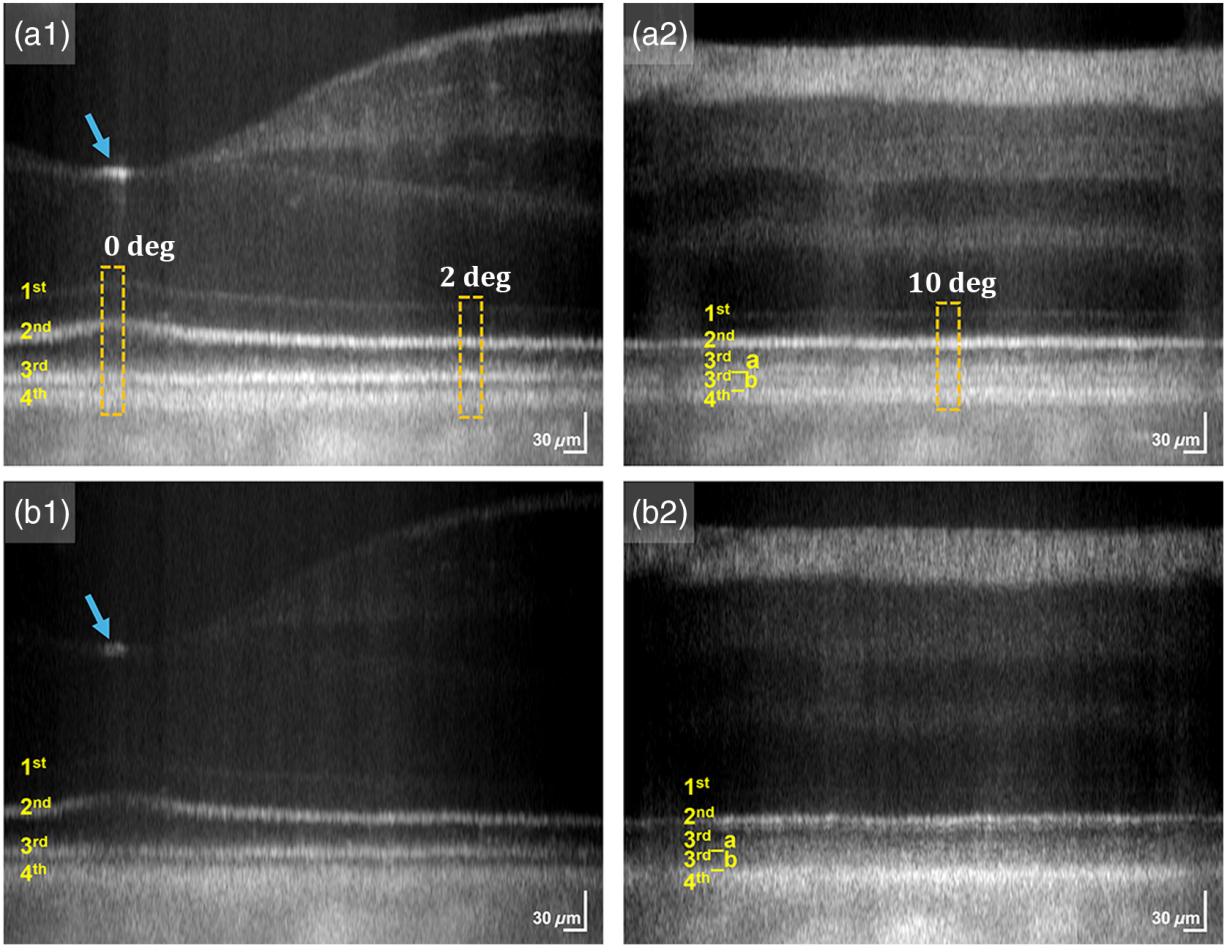
Representative (a) parallel and (b) cross-polarization OCT mean B-scans displayed in logarithmic scale, obtained to record [(a1), (b1)] 0- and 2-deg nasal eccentricities, and [(a2), (b2)] 10-deg nasal eccentricity. Data were recorded from subject 1 in light conditions. The blue arrow in (a1) and (b1) denotes the central foveal reflection pattern. The 1st, 2nd, 3rd, 3rd_a, 3rd_b, and 4th outer retinal band corresponds to the external limiting membrane (ELM), inner segment/outer segment (IS/OS) junction or ellipsoid zone (EZ), outer segment tip (OST) or interdigitation zone (IZ), cone outer segment tip (COST) or cone interdigitation zone (CIZ); rod outer segment tip (ROST) or rod interdigitation zone (RIZ), retinal pigment epithelium/Bruch’s membrane (RPE/BM) complex, respectively.

The OCT volumes were reconstructed from the raw data using MATLAB (MathWorks, Inc., Natick, Massachusetts, United States). The average of all the volumes was obtained first and then subtracted from each volume to eliminate fixed phase-stable artifacts. Computational dispersion compensation was applied to correct spectral distortions. After dispersion compensation, the data were five-fold zero-padded, enhancing axial pixel sampling resolution to 0.58  μm. OCT volumes were reconstructed using the Fourier transform, and four OCT volumes with a high signal-to-noise ratio were obtained from the same retinal location. These four volumes were registered using a 3D temporal speckle averaging registration algorithm.^43^^,^^44^ To maintain consistency during volumetric registration, we utilized a reflection pattern from Henle’s fiber layer (HFL) as an image marker (shown in Fig. 2) for the registration and alignment of 0- and 2-deg nasal eccentricities data. This pattern, arising from the oblique orientation of the foveolar photoreceptor axons along the OPL,^45^ has been employed in laser polarimetry^46^ for accurately pinpointing the foveal center. In Fig. 2(a), the enface image derived from a whole-depth projection lacks this reflection pattern, making it difficult to identify the foveal center. However, the bright reflection pattern was distinctly visible in Fig. 2(b), where the enface image was obtained from the inner retinal projection, spanning from the IPL to the HFL. To further verify this central foveal reflection pattern, Figs. 2(c1) and 2(c2) show averaged B-scan images corresponding to yellow and orange window regions in Fig. 2(b). The center of this reflectance pattern aligns with 0-deg nasal eccentricity, serving as a unique marker for precise image registration across different recordings. The same pattern was consistently observed across consecutive volumes, serving as a reliable marker for transverse registration. Although minor variations in the reflection pattern may occur due to instrument orientation and alignment, the center of this pattern remained highly consistent in both light- and dark-adapted OCT images and was used as a reference marker in our analysis. For the data acquired at 10-deg nasal eccentricity, the reflection pattern from the nerve fiber layer was utilized as the landmark for registration and alignment. Thus, the central fovea reflectance pattern in enface OCT [Fig. 2(b)] could be used for image registration in the transverse dimensions, whereas the layer structure in B-scan [Fig. 2(c)] enables image registration in the axial direction, and this 3D volumetric registration facilitates reliable comparative analysis of OCT recordings acquired under light and dark conditions. After image registration, four consecutive volumes were averaged to produce a mean volume. This volume was then flattened relative to the RPE/BM layer, and a mean B-scan was generated by averaging 30 adjacent B-scans from the flattened dataset for comparative analysis. Ensuing B-scan averaging, 30 adjacent A-lines from specific locations of the mean B-scan (as shown in Fig. 3) were averaged to compute the mean axial intensity profiles. The multi-step averaging process significantly reduced noise and improved the signal-to-noise ratio of the reflectance profiles, ensuring more reliable results. To further refine the profiles, we applied nine-fold cubic interpolation, enhancing resolution for detailed analysis of the outer retinal bands. Peak locations were identified using the peak-finder function in OriginPro software and manually cross-validated to eliminate false detections. The statistical significance of thickness alterations in the retinal bands was assessed using a paired-sample t-test at a 95% confidence level. We have assessed the significance of the thickness alterations with P-values of 0.05, 0.005, and 0.001, where 0.001 indicates higher significance than 0.05. Data analysis workflows were executed in MATLAB and ImageJ, whereas statistical evaluations were performed using OriginPro software (Version 2023, OriginLab Corporation, Northampton, Massachusetts, United States).

## 3 Results

**Fig. 4:**
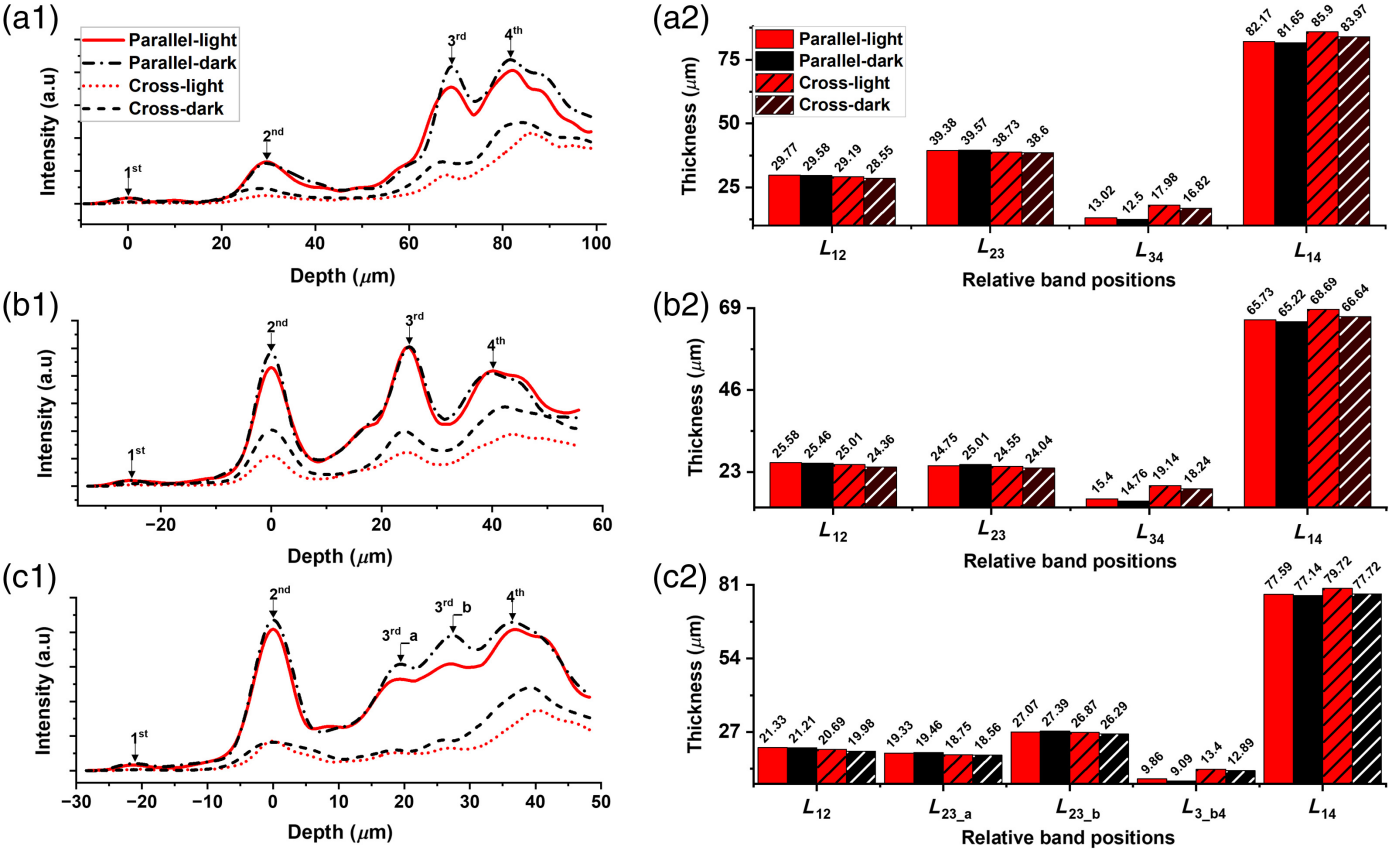
Representative axial intensity profiles (a1), (b1), and (c1) and corresponding band distances (a2), (b2), and (c2) obtained from light- and dark-adapted retinas using parallel and cross-polarization OCT. Data were recorded from subject 1 at (a) 0-deg nasal eccentricity, (b) 2-deg nasal eccentricity, and (c) 10-deg nasal eccentricity.

Parallel-polarization and cross-polarization OCT measurements were conducted under light and dark-adapted conditions. Representative parallel-polarization OCT B-scans obtained during the light condition are presented in Fig. 3 with a logarithmic scale. In Fig. 3, the first to fourth outer retinal bands correspond to the external limiting membrane (ELM), the IS/OS junction or EZ, the OST or IZ layer, and the RPE/BM complex, respectively. In addition, the bands labeled 3rd_a and 3rd_b in Fig. 3(b) represent the cone outer segment tip (COST) or cone interdigitation zone (CIZ) and the rod outer segment tip (ROST) or rod interdigitation zone (RIZ). To enhance the visualization of detailed retinal structures, we presented the B-scans on a logarithmic scale (Fig. 3). Although this approach improves contrast, it also exaggerates the widths of hyperreflective layers, potentially distorting the accurate thickness estimates of various outer retinal layers.^47^ This distortion could lead to inaccurate comparative assessments as the band thicknesses may appear larger than they are. Therefore, axial intensity profiles used for quantitative OCT band analysis were based on linearly scaled values. Given the distinct functional contributions of rod and cone photoreceptors in light and dark-adapted conditions, we recorded OCT data from three regions with varying densities of these photoreceptors. The orange rectangles in Figs. 3(a) and 3(b) demarcate these regions. The first region, recorded at 0-deg nasal eccentricity [Fig. 3(a)], represents a cone-only area. The second region, at 2-deg nasal eccentricity [Fig. 3(a)], includes both rods and cones, with the rod density being 1.2 times higher than the cone density.^48^ The third region, recorded at 10-deg nasal eccentricity [Fig. 3(b)], is rod-dominant, with rod density being 12 times greater than cone density.^48^ We averaged 30 adjacent A-lines from each demarcated region to obtain mean axial intensity profiles. Figure 4 illustrates representative parallel-polarization and cross-polarization OCT profiles obtained under light and dark-adapted conditions. All axial intensity profiles were aligned to the location of the first outer retinal band, the ELM. In Fig. 4, the distance between the first and second outer retinal bands is labeled as $L_{12}$. Similarly, $L_{23}$, $L_{34}$, and $L_{14}$ denote the distances between the second and third; third and fourth; and first and fourth outer retinal bands, respectively. In the rod-dominant region [Fig. 4(c)], the distances between the second and 3rd_a; second and 3rd_b; and 3rd_b and 4th bands are labeled as $L_{23\_a}$, $L_{23\_b}$, and $L_{3\_b4}$, respectively.

The inner segment (IS) length ($L_{12}$) increased under light conditions compared with the dark-adapted stage, a trend observed consistently across all three regions [Figs. 4(a)–4(c)]. Using parallel-polarization OCT, light-induced swelling of $L_{12}$ was measured to be 0.19, 0.12, and 0.12  μm at 0 deg [Fig. 4(a2)], 2 deg [Fig. 4(b2)], and 10 deg [Fig. 4(c2)] nasal eccentricities, respectively. A similar trend was observed in cross-polarization OCT measurements obtained at 0 deg [Fig. 4(a2)], 2 deg [Fig. 4(b2)], and 10 deg [Fig. 4(c2)] nasal eccentricities demonstrating increases of 0.64, 0.65, and 0.71  μm in $L_{12}$, respectively. Notably, the largest light-induced elongation of $L_{12}$ with parallel-polarization OCT was observed in the cone-only region [Fig. 4(a2)], whereas the highest increase recorded by cross-polarization OCT was in the rod-dominant region [Fig. 4(c2)].

The band distance $L_{23}$, i.e., the OS length, appeared to shrink under light [Figs. 4(a)–4(c)]. Parallel-polarization OCT revealed that $L_{23}$ was consistently shorter in light-adapted conditions compared with the dark-adapted state across all three regions. Specifically, $L_{23}$ decreased by 0.19  μm at 0 deg [Fig. 4(a2)] and 0.26  μm at 2 deg [Fig. 4(b2)] nasal eccentricities. The most significant light-induced shrinkage, however, occurred at the rod-dominant 10-deg eccentricity [Fig. 4(c2)], where $L_{23\_b}$ shortened by 0.32  μm, whereas the shrinkage for $L_{23\_a}$ was 0.13  μm. By contrast, cross-polarization OCT did not detect any light-induced shrinkage in $L_{23}$. Instead, it showed an increase in $L_{23}$ under light-adapted conditions across all regions, with swelling of 0.13  μm at 0 deg [Fig. 4(a2)], 0.51  μm at 2 deg [Fig. 4(b2)], and 0.19 and 0.59  μm ($L_{23\_a}$: 0.19  μm and $L_{23\_b}$: 0.59  μm) at 10 deg [Fig. 4(c2)] nasal eccentricities.

The subretinal space is the distance between the third band OST and fourth band RPE/BM complex ($L_{34}$), which appeared to increase in light conditions [Figs. 4(a)–4(c)]. Light-evoked swelling of the subretinal space was evident in both parallel and cross-polarization OCT. Parallel-polarization recording revealed a maximum swelling of 0.76  μm at 10 deg [Fig. 4(c2)] nasal eccentricity ($L_{3\_b4}$) followed by 0.64  μm at 2 deg [Fig. 4(b2)] and 0.52  μm at 0 deg [Fig. 4(a2)] nasal eccentricity. For cross-polarization OCT, the light-induced swelling at 0 deg [Fig. 4(a2)], 2 deg [Fig. 4(b2)], and 10 deg [Fig. 4(c2)] nasal eccentricities was 1.16, 0.9, and 0.51  μm respectively.

The outer retinal thickness ($L_{14}$) increased in the light-adapted stage [Figs. 4(a)–4(c)]. In parallel-polarization OCT, the outer retina elongated by 0.52, 0.51, and 0.45  μm at 0 deg [Fig. 4(a2)], 2 deg [Fig. 4(b2)], and 10 deg [Fig. 4(c2)] nasal eccentricities, respectively. When the outer retinal thickness changes were examined using cross-polarization OCT, a more significant light-driven swelling was noted at all imaging positions, with an elongation of 1.93  μm at 0 deg [Fig. 4(a2)], 2.05  μm at 2 deg [Fig. 4(b2)], and 2  μm at 10 deg [Fig. 4(c2)] nasal eccentricities.

**Fig. 5:**
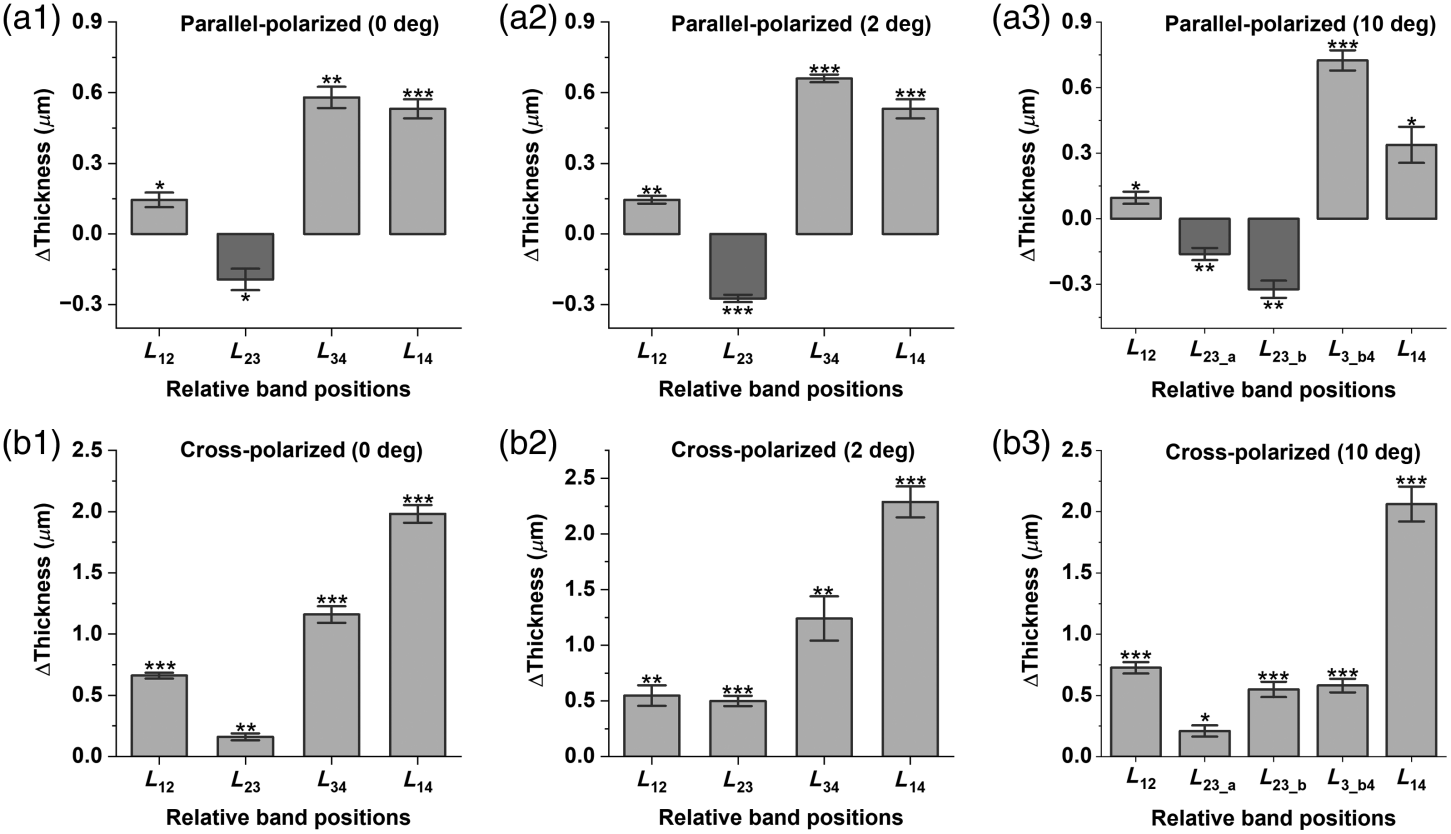
Mean and standard error plot of thickness differences between light- and dark-adapted retinas using (a) parallel-polarization and (b) cross-polarization at [(a1), (b1)] 0-deg nasal eccentricity, [(a2), (b2)] 2-deg nasal eccentricity, and [(a3), (b3)] 10-deg nasal eccentricity. Each value was estimated by subtracting the thickness in dark conditions from the thickness in light conditions. Statistical significance was conducted using a paired sample t-test with a confidence level of 95%. P(0.05) = *; P(0.005) = **; P(0.001) = ***.

To assess the repeatability of our findings, we calculated the mean and standard error of the thickness changes obtained from all four healthy subjects during the transition from light to dark conditions. These values are illustrated in Fig. 5. Statistical significance was evaluated using a paired sample t-test, as detailed in Sec. 2. The $L_{12}$ thickness changes revealed in parallel-polarization OCT were 0.14, 0.15, and 0.09  μm at 0 deg [Fig. 5(a1)], 2 deg [Fig. 5(a2)], and 10 deg [Fig. 5(a3)] nasal eccentricities, respectively. In cross-polarization, these values were notably larger: 0.66  μm at 0-deg [Fig. 5(b1)], 0.54  μm at 2-deg [Fig. 5(b2)], and 0.72  μm at 10-deg nasal eccentricity [Fig. 5(b3)]. A light-induced elongation of the photoreceptor IS was commonly observed in both modalities, with more pronounced changes in cross-polarization OCT. For the photoreceptor OS ($L_{23}$), light-driven shrinkage was noted in parallel-polarization OCT, with mean values of −0.19  μm [Fig. 5(a1)] and −0.27  μm [Fig. 5(a2)] at 0- and 2-deg nasal locations, and −0.16  μm ($L_{23\_a}$) [Fig. 5(a3)] and −0.32  μm ($L_{23\_b}$) [Fig. 5(a3)] at 10-deg nasal locations. Conversely, cross-polarization OCT revealed OS elongation, with mean values of 0.16  μm [Fig. 5(b1)], 0.49  μm [Fig. 5(b2)], 0.21  μm ($L_{23\_a}$) [Fig. 5(b3)], and 0.55  μm ($L_{23\_b}$) [Fig. 5(b3)] at 0-, 2-, and 10-deg nasal eccentricities. For parallel-polarization OCT, subretinal space ($L_{34}$) swelling was 0.58  μm [Fig. 5(a1)], 0.66  μm [Fig. 5(a2)], and 0.72  μm [Fig. 5(a3)] at 0-, 2-, and 10-deg nasal eccentricities, respectively. In cross-polarization OCT, subretinal space swelling was more pronounced (apart from 10 deg), with mean values of 1.16  μm [Fig. 5(b1)] at 0 deg, 1.24  μm [Fig. 5(b2)] at 2 deg, and 0.58  μm [Fig. 5(b3)] at 10 deg. The parallel and cross-polarization recordings revealed an overall increase in outer retinal length ($L_{12}$) at all three locations. The mean increase in $L_{12}$ thickness was detected to be 0.53  μm [Fig. 5(a1)], 0.54  μm [Fig. 5(a2)], and 0.33  μm [Fig. 5(a3)] at 0, 2, and 10 deg, respectively, with subretinal space swelling contributing significantly, particularly in the rod-dominant regions. Cross-polarization OCT revealed significant elongation under light, with mean values of 1.98  μm [Fig. 5(b1)], 2.18  μm [Fig. 5(b2)], and 0.58  μm [Fig. 5(b3)] at the respective eccentricities. All thickness changes were statistically significant at a 95% confidence level according to the paired sample t-test, as illustrated in Fig. 5.

## 4 Discussion

In summary, this study employed polarization-resolved FF-SS-OCT to investigate dynamic changes of the human photoreceptor OS and subretinal space under light and dark-adapted conditions. Comparative parallel-polarization and cross-polarization OCT measurement confirmed light-driven OS shrinkage and subretinal space expansion.

Light-induced swelling in overall outer retinal length was consistently observed in both parallel-polarization and cross-polarization OCT. The outer retinal thickening is characterized by increased distance between the first band ELM to the fourth band RPE/BM complex ($L_{14}$). Our observation aligns with findings from previous studies in both human and animal models where the light-dependent outer retinal swelling has been attributed to phototransduction-initiated osmotic swelling and increased hydration of subretinal space.^17^^–^^19^^,^^49^

Parallel-polarization OCT revealed a reduction of photoreceptor OS length in light-adapted retinas. The ROST and COST tips were individually identified in the rod-dominant region, where OS shrinkage was more pronounced in rods than cones. Reduction of OS length was detected reliably using parallel-polarization OCT, which primarily detects ballistic photons supposedly originating from layered structures such as IS/OS junction and OST. The photoreceptor OS length has been estimated as the distance between these two layers ($L_{23}$), enabling parallel-polarization OCT to offer better sensitivity in detecting OS. By contrast, cross-polarization OCT data are dominated by multiply scattered photons from scattering tissue structures such as the EZ, IZ, and RPE/BM complex layers. The resultant cross-polarization data showed light-induced swelling of OS, likely to represent the dynamics of highly scattering EZ and IZ zones. The presence of such multiply scattered photons makes it difficult to detect IS/OS junction and OS tips reliably, consequently affecting the reliable monitoring of OS dynamics.

The light-induced OS shrinkage observed in our parallel-polarization OCT data contrasts with reports from traditional OCT systems, which have predominantly reported light-induced OS swelling.^17^^–^^19^ However, it must be noted that traditional OCT-based experiments’ time scales differed from ours. Light-induced shrinkage of photoreceptors OSEL reported in healthy human subjects was observed after 10 to 20 min of continuous light exposure in dark-adapted retinas. This study,^15^ using a commercial OCT system, reported a mean shrinkage of 2.14  μm, which is significantly higher than the parallel-polarization shrinkage observed in our study. This discrepancy is predominantly due to different definitions of the OS length. The previously reported OSEL was defined as the region from photoreceptor OS to the inner boundary of RPE apical processes, which includes both OS length and subretinal space between the OS tip and RPE. In our experiment, the OS was defined as the length from the IS/OS junction to the OS tips, and subretinal space was referred to as the distance between the OS tip and RPE. When the photoreceptor OS length was estimated between the IS/OS and ROST/COST layer, an oscillatory change over time was recorded as a response to flash stimuli.^18^ The initial elongation (peaked around 5 to 10 min of post-bleaching) reports a change opposite to what we have observed in parallel-polarization OCT. However, the shrinkage of OS length around 20 to 30 min is consistent with our observation. Such oscillatory change suggests that OS length alteration follows complicated dynamics, can be time-dependent, and is likely to have different contributing factors for the reported biphasic mechanisms.

In conventional OCT systems, fiber-based polarization controllers can be used to match the polarization states of the sample and reference arm signals. If the illumination light is linearly polarized, the detected OCT signal is likely to be dominated by reflection signals with polarization in the direction of the illumination light. However, if the illumination light is not linearly polarized, the OCT will not be able to differentiate the directly reflected photon with preserved polarization condition from multiply scattered photons with depolarization. Therefore, a nonuniform variation of the polarization matching across the wavelengths will lead to the distortion of the point spread function at different depths.^50^ This phenomenon is known to affect axial resolution. Moreover, the detector plane typically lacks any active polarization control; thus, the polarization state of the interference signal will be altered by the optical fiber itself along the detector plane. Hence, even though the detected signal is dominated by the reflection signal, it would not be able to maintain the resolved polarization state entirely. In our polarization-resolved FF-SS-OCT system, we utilize a combination of linear polarizer, QWP, and a free space optical system to ensure precise detection of parallel and cross-polarization signals. The lack of a polarization-resolved approach in conventional OCT systems leads to the capture of both ballistic and multiplying scattered photons, complicating the reliable detection of the IS/OS junction and OS tip. This discrepancy highlights the importance of using polarization-resolved imaging to accurately monitor outer retinal dynamics as traditional systems may conflate changes in different retinal layers. For quantitative comparison, we repeated our light-dark adaptation experiment without using any polarization control, i.e., the linear polarizer and the QWP were removed during the data collection. Subject 2 was imaged using the same protocol, as described in Sec. 2. Comparative assessment between the different band positions revealed no significant difference in $L_{12}$ at all three positions. $L_{23}$, which represents OS length, only showed notable shrinkage at 10 deg ($L_{23\_a}$: 0.06  μm, $L_{23\_b}$: 0.29  μm), which was lower than the shrinkage observed for parallel-polarization data [Fig. 5(a)]. The only significant change that we observed was in $L_{34}$, which was reported to show light-driven swelling of 0.25, 0.32, and 0.51  μm at 0-, 2-, and 10-deg nasal eccentricities, respectively.

Further complications in quantifying OS dynamics can arise from the conflicting boundary definition. For instance, the light-driven thickening reported in previous experiments^17^^,^^19^^,^^34^ considered the distance between ELM and RPE or between IS/OS and RPE while monitoring the OS dynamics. We observe similar light-induced swelling in $L_{14}$ and $L_{24}$ (distance between the second band IS/OS to the fourth band RPE/BM complex) as the swelling in subretinal space ($L_{14}$) is higher than the shrinkage in OS ($L_{23}$). However, the IS ellipsoid or RPE/BM complex is not a structural component of the OS itself. Considering changes between these layers while monitoring OS dynamics might lead to inaccurate estimations.

This light-driven OS shrinkage observed in parallel-polarization may be attributed to reduced spaces between disc membranes within the OS, a phenomenon previously observed in microscopic studies.^38^ This change was observed in isolated rods and intact retinas. We can hypothesize that the inter-disk space reduction under light contributed to the OS shrinkage observed in our study, whereas the osmotic swelling contributed to subretinal space expansion. The increased OS length in cross-polarization OCT might reflect the changes in the EZ and IZ zones, along with potential complexity in peak estimation. Phase-resolved OCT^16^^,^^25^^,^^32^ studies have shown an initial shrinkage (∼20 to 50 nm) followed by swelling of OS (∼200 to 400 nm) within a few seconds of bright light stimulation, estimated from optical path length differences between two layers. Although the sensitivity of the phase-resolved technique can be compromised when averaging phase data across extended areas, the impact of multiply scattering in phase estimations needs to be assessed.^28^ Simultaneous monitoring of OCT amplitude and phase data during light-to-dark transitions may provide deeper insights into the relationship between structural changes and optical path length variations in functional imaging.

## 5 Conclusion

A comparative assessment using parallel-polarization OCT of light- and dark-adapted retinas across varying visual field eccentricities confirmed light-induced shrinkage of the photoreceptor OS, accompanied by subretinal space swelling. In cross-polarization OCT, multiply scattered photons dominate, complicating the reliable detection of OS dynamics in both light- and dark-adapted retinas. Polarization-resolved OCT promises an improved ORG modality for early detection and treatment assessment of eye diseases.

## Data Availability

Data supporting the findings of this study are available from the corresponding author upon reasonable request.
